# Supplementary material for: Multidrug-resistant E. coli encoding high genetic diversity in carbohydrate metabolism genes displace commensal E. coli from the intestinal tract
Source: PLoS Biol. 2023 Oct 17;21(10):e3002329. doi: 10.1371/journal.pbio.3002329 (PMC10581457; doi:10.1371/journal.pbio.3002329)
Supplement: S1 Method — (DOCX) [file pbio.3002329.s001.docx]

Supplementary Methodology

Histological and cytokine analysis

Sections of mouse gut were preserved in RNAlater Stabilization solution (Thermo) before homogenization in lysis buffer. Tissues were homogenised in RLT buffer (Qiagen) using a stainless-steel bead and agitating in a FastPrep-24 (MPBio) in 3x1 minute bursts at a speed setting of 6.0m/s with 1 minute intervals on ice. The resulting lysate was used as input for the RNeasy Mini kit (Qiagen) including an on-column DNase treatment (Qiagen). Extracted RNA was converted to cDNA using High-Capacity cDNA Reverse Transcription kit (Applied Biosystems) with random primers. Pre-designed probes for qPCR were obtained from Integrated DNA Technologies (IDT –S4 Table) and reactions were performed using PrimeTime Gene Expression master mix (IDT) on a QuantStudio 1 system (Thermo). Reactions were performed following manufacturer’s recommended parameters: 3 minutes at 95*C, 40 cycles of 15 seconds at 95*C, 1 minute at 60*C, fluorescence readings taken at the end of the extension stage. Cytokine Ct values were normalised to the endogenous Pol2ra control and presented as deltaCt values. Significance was determined using 2-way ANOVA with Tukey’s multiple correction.

Sections of mouse gut were ‘swiss-rolled’, 10 % formalin fixed, dehyrated and embedded in paraffin. Tissues were sectioned longitudinally, mounted onto glass slides and stained with haematoxylin and eosin. Slides were imaged using an Axioscan 7 Slide Scanner (Zeiss) and blind scored using Fiji following the scoring system in^1^.

Number of fields of view scored for each condition

| Colonisation Condition | | Small Intestine | Colon |
| --- | --- | --- | --- |
| Monocolonised | 822-E8 (n=3) | 26 | 19 |
|  | F084 (n=3) | 12 | 18 |
|  | F016 (n=3) | 19 | 15 |
| Co-inoculated / Competitive Colonisation | 822-E8 & F084 | 15 | 12 |
|  | 822-E8 & F016 | 12 | 12 |
|  | F084 & F016 | 12 | 12 |
| Monocolonised with challenge after 7 days | 822-E8 challenged with PBS | 12 | 15 |
|  | 822-E8 challenged with F084 | 15 | 15 |
|  | 822-E8 challenged with F016 | 12 | 12 |
|  | F016 challenged with 822-E8 | 12 | 12 |
| Germ Free | Germ Free | 21 | 21 |

**References**

1. Erben, U., Loddenkemper, C., Doerfel, K., Spieckermann, S., Haller, D., Heimesaat, M. M., Zeitz, M., Siegmund, B., & Kühl, A. A. (2014). A guide to histomorphological evaluation of intestinal inflammation in mouse models. *International Journal of Clinical and Experimental Pathology*, *7*(8), 4557–4576.
