# Supplementary figures and images for: Multidrug-resistant E. coli encoding high genetic diversity in carbohydrate metabolism genes displace commensal E. coli from the intestinal tract
Source: PLoS Biol. 2023 Oct 17;21(10):e3002329. doi: 10.1371/journal.pbio.3002329 (PMC10581457; doi:10.1371/journal.pbio.3002329)

**A**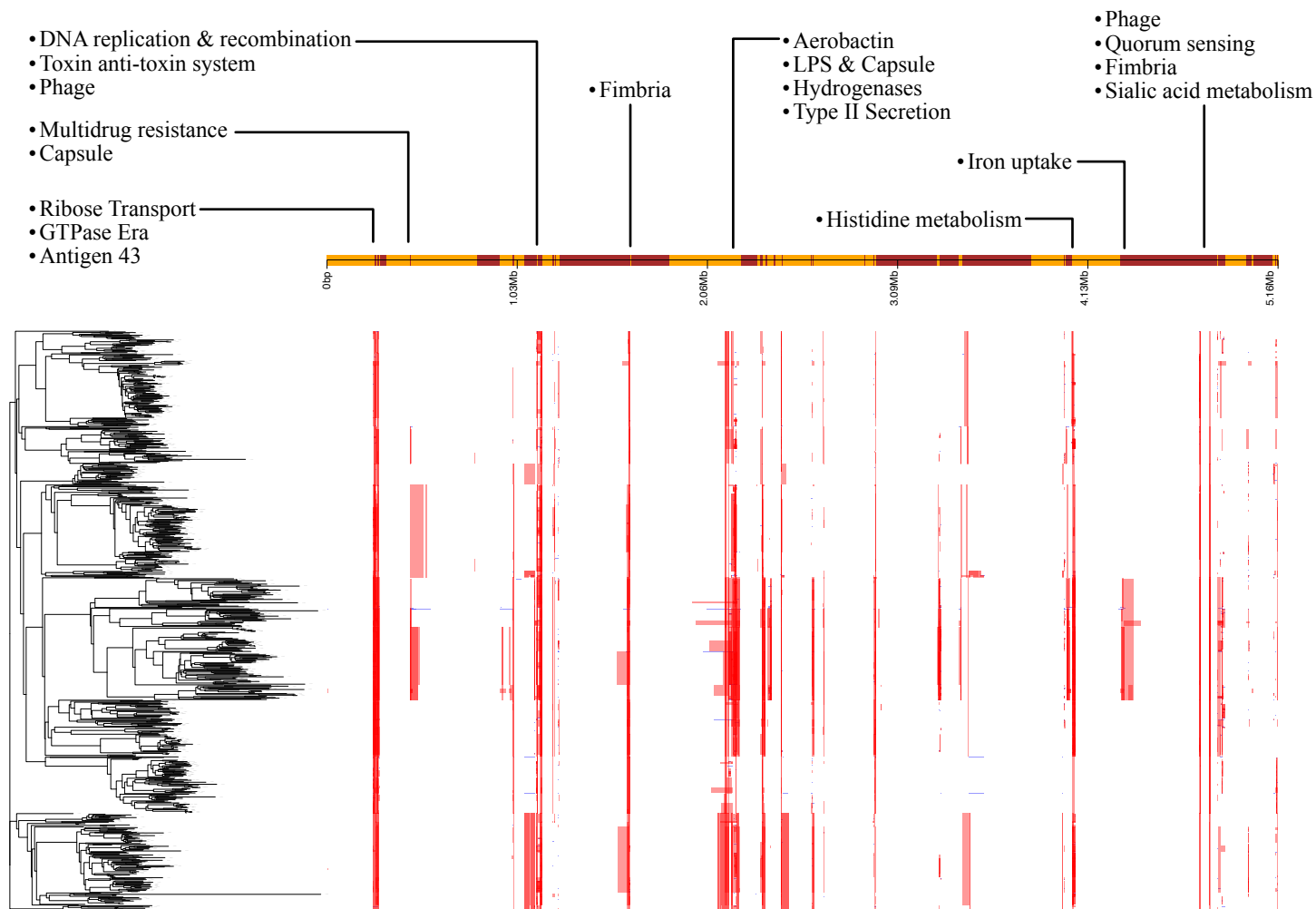**B**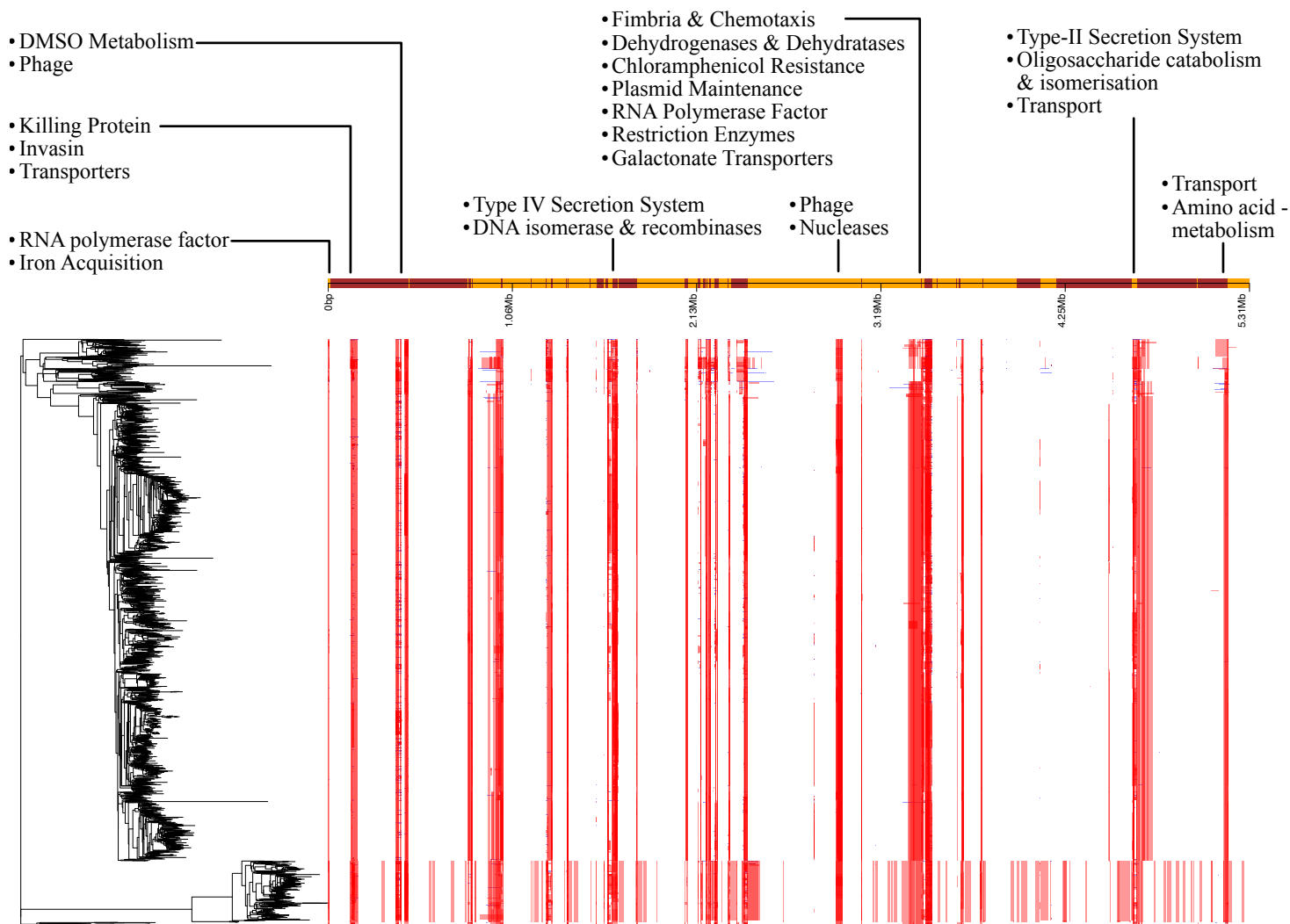

Supplement: S18 Fig — Recombination regions predicted by Gubbins in ST73 (A) and ST131 (B) lineages. Red blocks indicate putative regions of recombination. Yellow and orange track bar indicate position in reference genome. Select regions are annotated. (PDF) [file pbio.3002329.s023.pdf]
