# Supplementary material for: Multidrug-resistant E. coli encoding high genetic diversity in carbohydrate metabolism genes displace commensal E. coli from the intestinal tract
Source: PLoS Biol. 2023 Oct 17;21(10):e3002329. doi: 10.1371/journal.pbio.3002329 (PMC10581457; doi:10.1371/journal.pbio.3002329)
Supplement: Attachment — Submitted filename: Response to reviewers.docx [file pbio.3002329.s036.docx]

Dear Dr Jauregui

We thank you for the comprehensive review of our manuscript, and your patience with us whilst we reflected on the best way to address the concerns relayed by the reviewers. Below we provide a point by point response to the comments. As you will see we do not believe all points raised can reasonably be addressed within the remit of this study, the experiments proposed would need to incorporate microbiome analysis, metagenomics and metabolomic profiling of microbial communities. While the reviewers requests seem simple they are in fact a considerable amount of work.

What we have done is incorporate new biolog analysis of the mouse competition strains and comparative genomic analysis of the three strains on the back of the reviewers comments which we believe now substantially strengthens and improves this paper and so we thank the reviewers enormously for their suggestions.

We hope we have provided a significantly revised and added to study which we hope will satisfy reviewers concerns and lead them to believe our study merits publication.

**Reviewer 1:**

*Major comments: (specific comments follow below)
1.      While the utilized germfree model is very simplistic and allows in vivo comparison of direct competition, I have my doubts how general the conclusions are in light of recent studies showing that commensal bacteria beyond the family Enterobacteriaceae contribute to competition between related bacteria in the gut (Eberl et al, Cell Host Microbe 2021 and Osbelt et al. Cell Host Microbe 2021). Moreover, in germfree mice, many colonization relevant carbohydrates are not released from dietary polysaccharides.*

We also agree that our findings now need to be further explored in the presence of a mature microbiome and indeed discuss this in line… However this was a first ever exploration of this displacement phenotype and for this exploratory study we explicitly decided to prove direct inter strain competition. We are confident our work will spark more detailed characteristic studies.

*2.      Very little evidence is provided that competition for carbohydrates and not differences in the ability to acquire other growth limiting factors such as amino acids and trace elements or the ability to utilize redox acceptors contribute to the competition in vivo. Therefore, how are the two analysis linked to each other?*

We agree with all three reviewers that this linkage of our data sets was lacking, We have now conducted a significant Biolog experiment and comparative genomics of the mouse competition strains. The data presented shows how valuable this suggestion has been and now demonstrates that in fact the allelic variation seen in MDR strains is most likely metabolic streamlining through gene loss via indels and mutations based on the exhaustive characterisation of our three competition strains. We are now re-analysing our 20K genomes for gene inactivation which will take some time but will be a following manuscript in the next 18-24 months.

*3.      The hypothesis of the authors are highly relevant, but they generalize their observations too much. Single strains are tested, which limits the conclusions that can be drawn. I would suggest that the authors should make this much clearer by referring to strains rather the STs throughout the different sections of the manuscript, including the abstract, results, and discussion.*

We now refer to strains in the mouse and biolog sections of the paper *Specific comments:
Line 142: should be "16S rRNA" There is also a sentence fragment in that line.*

Now corrected

*Line 227ff: Make sure to refer to specific strains rather than STs. In general, throughout the whole study, just one strain was used per condition (MDR, non-MDR, commensal). To see whether the effect is strain specific or if it's a general phenotype that MDR ExPEC strains outcompete commensal strains, experiments should be repeated with different strains of each category.*

We now name the strains in the mouse and biolog sections by strain name. We agree that more exhaustive experiments will be needed for general interpretation, however, given the extensive and expensive nature of these experiments, we do believe that the current work provides sufficient evidence and novelty to merit a publication.

*Line 229ff: In general the authors do not give a comprehensive description of the strains… Also, if carbohydrate utilization is the key factor, why do not test growth in different carbon sources for those three strains? What are the differences in carbohydrate preferences? Can they equally grow in important sugars available in the gut of mice (e.g sucrose, glucose, cellobiose etc.). The statement of "carbohydrate utilization" is subsequently very broad and not really followed on in the whole study? By growing strains in different media and monitoring the general carbohydrate utilization capability, it would be at least possible to identify a group of potential candidate sugars.*

We now provide a comprehensive Biolog aganlysis of the three strains and detailed targeted comparative genomics to identify the key differences in metabolism between the three strains.

*Line 239f: Data shown in FigS3 shows the colonization levels of a mono-colonization with the strains. This data shows, that the MDR ExPEC strain shows 5-10 fold higher colonization levels in germfree mice at day 1-5. This could influence the following phenotypes. Could strains with similar colonization levels be used? If no extra experiments are performed, please discuss this limitation.*

We concede that the ST131 strain does show initial higher colonisation levels in mono-colonised mice despite mice being gavaged with an equal CFU for every experiment. Nevertheless, we believe this initial higher colonisation level does not explain the ability of the ST131 strain to prevent an ‘invading’ commensal strain from establishing itself in the mouse (both when introduced via gavage and co-housing), particularly as these experiments were performed at day 7. We believe that this demonstrates that the ST131 strain assayed is out-competing the commensal strain.

*Line 241ff: It is interesting that apparently the strains do replace each other in the gut. What happens in a defined community of strains? It is rather unlikely that travelers harbor no community in the gut when acquiring new E. coli strains. Is this working in a similar manner, when mice with low or high complex communities are used? E.g. are the E. coli strains naturally colonizing the mice from different vendors also replaced by the superior E. coli strains?*

This is a very interesting question but we believe is the focus of a follow on study. As stated above this was a first ever exploration of this displacement phenotype and for this exploratory study we explicitly decided to prove direct inter strain competition. We are confident our work will spark more detailed characteristic studies.

*Line 248 ff. Are the strains predicted to be capable to produce toxins and phages? If so, is LB a sufficient medium to induce lytic prophage induction and release of toxins? In other enterobacterial species, toxin production is tightly regulated (e.g induction for specific substrates like sugar or iron). I would suggest, to include other modified media to exclude that toxins or phages are relevant.*

ST131 do not encode a functional T6SS (<https://doi.org/10.1101/2023.03.28.534550>) and whilst there are phages in the genome it is impossible to know if these are lytic or not. Given that we see a retardation of growth of non-ST131 by ST131 in LB it is reasonable to assume we would show if this was phages or toxins using the method presented here.

*If the MDR strains encode lytic prophages, it would be a clearer experiment to generate phage deficient strains and test, whether those strains fail to replace the commensal strain in vivo. Also, plaque-formation in the commensal bacteria with intestinal content of MDR-colonized could be evaluated to see whether in vivo something different happens.
In a similar direction: what happens, if strains are co-cultured in minimal media with different carbon sources? Do strains co-exist or is the commensal strain equally outcompeted? It would be at least crucial to prove in simplified in vitro experiments that the strains do outcompete each other for different substrates.*

Whilst these experiments are of course of enormous value we believe they are way outside the remit of this study and would require years of molecular biology and in vivo studies which we hope will be the focus of studies following this publication. We point the reviewer to our preprint (<https://doi.org/10.1101/2023.03.28.534550>) which shows retardation in simple media and which we now cite.

*Line 293ff: : The data are largely descriptive, and none of the observations is followed on mechanistically. The mice used in this study are raised germfree, so its absolutely obvious that challenge with any kind of bacteria will stimulate the immune system. Different cytokines were measured to "assess host mucosal immune response" and the author claim relatively few differences between colonization conditions. Does that mean, that the immune response is dispensable? I don't understand, why this data are included in the manuscript. If the focus is shifted to potential differences in the immune system, the authors should provide more detailed data than one histology picture and levels of 11 cytokines.  Specifically, line 307ff "Collectively, these data demonstrate that displacement of commensal E. coli and dominant colonization by MDR ExPEC is associated with a distinct host mucosal immune response." I think, the few data on cytokine profiles do not support a statement like this…*

In light of the comment above, and the substantial addition of the biology and comparative genomics work we agree the cytokine work is preliminary at best. We have now reduced this toa descriptive paragraph and supplementary figures.

*Line 355ff: The authors identify an "increased number of carbohydrate metabolism and transport" genes. 
Does this translate in an increased ability of the MDR strains to utilize different carbohydrates? I suggest analyzing the three isolates using the Biolog Screening System to verify that the MDR strains indeed utilize a more diverse set of carbohydrates.*

This is an excellent suggestion and we have now incorporated Biolog data which shows a critically important observation that the alleles are in fact detrimental changes leading to metabolic streamlining

*Where specific carbohydrates identified as being most prominent in MDR strains? Are these carbohydrate utilization genes relevant in the mouse intestine? 
What happens when carbohydrate levels are increased in the mouse gut? If the carbohydrate utilization is the major factor of intraspecies competition, an increased amount of carbohydrates available in the mouse gut should impact the phenotype. This could be done by a supplementation with specific csrbohydrates, e.g., ones that are well/solely utilized by the commensal strains.*

Again this is an excellent suggestion but we believe is the subject of proper designed investigation following on from this first ever observation, especially now we have targeted substrates now from the Biolog data to pursue.

 **Reviewer 2:**

*This is a compelling study that begins to investigate how ExPEC MDR isolates behave in the host compared to non-MDR isolates. The study is significant because it begins to add a much needed layer of better understanding the physiology of these pathogens as opposed to merely looking at genomic content. 
I have a few questions/comments for the authors to consider:
1) The concept of altered metabolism is extremely interesting, but not discussed or expanded at all in the current study. This results section should be expanded and validated. Are there specific metabolism gene in CHO metabolism that are different? Are there more of them? What if a subset was transplanted to a non-MDR ExPEC strain? I may have missed this, but if the analysis was confined to the ExPECs, would this difference in metabolic gene carriage still be the same.*

This is an excellent question. We are now undertaking detailed gene by gene analyses of the pangenome data which is a lengthy process but will be submitted in the next 18 months or so. In the meantime we present a detailed comparative genomic analysis of the three competed strains to provide more detail and show that this is actually metabolic streamlining in the ST131. The idea of transplanting ST131 alleles is excellent but we consider it to be better placed in a follow up study. *2) Gnotobiotic mouse experiment: If the MDR strain is cured (to create an isogenic non-MDR clone), would the advantage persist? These studies are elegant, but may be worth repeating in a conventionally reared mouse strain that lacks Enterobacteriaceae to observe the interaction of the strains with the rest of the microbiota. finally, given the emphasis on ExPEC, the authors should evaluate transition of the strains to the bladder by performing longitudinal urinalysis. It would be very interesting to see if the MDR strain outcompetes in transmission as well.*

Again these are excellent suggestions of further work but we believe are outwith the scope of this specific study which is a first ever description and characterisation of the displacement phenotype. The work suggested here is excellent and logical follow on research.


**Reviewer 3:**

*Major comments:
-       Line 246: I was surprised that the ExPEC strains coexisted for 6 days, since I assume there are substantial differences. The authors should discuss the genomic differences between these two strains.*

We now provide a detailed comparative genomic analyses of the competition strains within the Biolog section

*Line 253: the growth curves in 1F-H do not rule out toxins or phage. Phage in particular are likely to be produced in times of stress that may not be mimicked by the in vitro growth environment but are present in the gut. Thus, more experiments are needed or this statement needs to be toned down a lot.*

We have now included caveats to our interpretation. However as pointed out above given that we see a retardation of growth of non-ST131 by ST131 in LB it is reasonable to assume we would show if this was phages or toxins using the method presented here

*-       Why could the non-MDR ExPEC strain not displace? This also seems like a worthy subject of at least discussion based on genomic content, and provides the substrate to generate candidates for knockout study in the MDR strain.*

We believe we make clear our hypothesis of why the MDR strain is unique in displacement. We now also provide biology and comparative genomic data to support our hypothesis

*-       Line 288: I don't see why the CFU equivalency means growth rate is the same.*

We now clarify our point in the text.

*-       Line 303: I don't understand why the hypothesis of carbohydrate metabolism would lead to the prediction of cytokine response and inflammation, as they observe?*

We now attempt to provide a clearer hypothesis based on that for other intestinal pathogens.

*-       Moreover, why is the inflammatory response only with gavage? Seems like it is something to do with initial colonization and adaptation. And if it only appears with gavage, seems like it is much less relevant to the typical human scenario, since we are not really gavaged with large quantities of pathogen.*

We of course do not wish to extrapolate to humans here. This is a first ever report of the observation of displacement and we anticipate will spark further characterisation studies.

*-       Line 336: the statement that AMR lineages are more recombinogenic but to the exclusion of phage seems to be implying much more mechanism than the correlation merits. Could easily be an indirect correlation. Also I don't see why it would be the proportion that matters - likely there is one or a few genes that enables metabolism of particular aspects of the diet that causes the fitness advantage in their strain.*

We show is a clear correlation between metabolism gene variation and AMR though there could of course be a number of factors that represent the true causal mechanism and the allelic variation and AMR are both only manifestations of those. We now caveat this though the fact that recombination also happens more in those lineages and has been shown to introduce alternative beneficial alleles in other studies such as in ST410 and ST167 merits the discussion in our opinion.

*-       Most of all, while I find the association of fitness with carbohydrate metabolism interesting, it is in some sense expected since previous evolution experiments in mice have shown that E. coli evolves by selecting for carb metabolism genes. Moreover, basing it on the correlation in Fig. 6 is highly speculative. For instance, they have only 3 strains from which to base their fitness idea in the first place - it could be that other MDR strains do not displace commensal E. coli!*

This is of course possible. What we provide here is compelling evidence of direct displacement based on inter-strain competition in a clean model. Given our other observations in people travelling to endemic countries we believe this is also a compelling case to propose to the community for future research.

*-       It would likely be straightforward to make a transposon library in this strain, which could then be screened for growth fitness, given the hypothesis of metabolism. This would provide a mechanistic test of their idea.*

The straightforward nature of making Tn libraries in real MDR strains could be debated at length. Regardless we believe this is a study in its own right and outwith the scope of this study reporting displacement for the first time. We hope the reviewer will be pleased to know we have received funding from BBSRC to do just these studies and will commence the work very soon. *Other comments:
-       The whole paragraph in the introduction ending on line 100 about recent studies of E. coli colonization seems like a series of anecdotes that could just be summarized in a single sentence, rather than going through all of the details.*

We believe these studies are instrumental in setting up our hypotheses and the study that follows. We have attempted to condense them into a more succinct story.

*-       Line 243: it would be interesting to provide some estimates of the fitness advantage of the ExPEC strains.*

We would not wish to conjecture on this without detailed fitness competition assay data, preferably in a properly designed in vivo model which seems superfluous to our displacement observation

*-       Throughout the manuscript in many places, the authors are not careful about verb tense. Many times things should be in past tense rather than the present that is in the manuscript - e.g. line 263.*

We have attempted to clear this up throughout the manuscript

*-       Line 333: why "incredibly"?*

We have now clarified this sentence for the reader
