# Supplementary material for: Multidrug-resistant E. coli encoding high genetic diversity in carbohydrate metabolism genes displace commensal E. coli from the intestinal tract
Source: PLoS Biol. 2023 Oct 17;21(10):e3002329. doi: 10.1371/journal.pbio.3002329 (PMC10581457; doi:10.1371/journal.pbio.3002329)
Supplement: Attachment — Submitted filename: final_response.docx [file pbio.3002329.s037.docx]

Dear Dr Jauregui

We thank you for the second appraisal of our manuscript. We of course are delighted at your offer to publish our paper as a discovery report and have made all the editorial edits requested in order to satisfy the criteria of this article type. The changes are clearly marked in the tracked changes document submitted and we hope these are to your satisfaction including the suggested chage to the title and dropping to four figures. We have also provided all the supporting raw data as supplementary files as requested. I can confirm no changes have been made to the reference list.

We are slightly disappointed that reviewer three feels our study requires a Tn-Seq experiment to merit publication. As mentioned in our previous rebuttal we have just received a 3 year BBSRC project to do this work, but that is the issue with the comment is that doing Tn-Seq and further in vivo experiments is a follow on stand on alone project. Clearly it will enhance our story enormously but we strongly believe it is not a revision request for the data presented here.

In response to reviewer 2, we would like to point out that we have in fact challenged the MDR ST131 strain with another phylogroup B2 strain. Our commensal gut isolate is a drug susceptible ST73 is a B2 strain and so the experiment has already been conducted. The suggestion to challenge two MDR strains is of course an excellent one but at the expense, time and need to use more mice we are unconvinced this data would either strengthen or weaken the conclusions we provide in the manuscript.

Many thanks for such a constructive process and we hope our changes will allow you to publish our work in PLOS Biology

Alan
